# ‘We are the eyes and ears of researchers and community’: Understanding the role of community advisory groups in representing researchers and communities in Malawi

**DOI:** 10.1111/dewb.12163

**Published:** 2017-09-05

**Authors:** Deborah Nyirenda, Salla Sariola, Kate Gooding, Mackwellings Phiri, Rodrick Sambakunsi, Elvis Moyo, Chiwoza Bandawe, Bertie Squire, Nicola Desmond

**Keywords:** Africa, bioethics, community advisory group or community advisory board, community engagement, community representatives, research

## Abstract

Community engagement to protect and empower participating individuals and communities is an ethical requirement in research. There is however limited evidence on effectiveness or relevance of some of the approaches used to improve ethical practice. We conducted a study to understand the rationale, relevance and benefits of community engagement in health research. This paper draws from this wider study and focuses on factors that shaped Community Advisory Group (CAG) members’ selection processes and functions in Malawi. A qualitative research design was used; two participatory workshops were conducted with CAG members to understand their roles in research. Workshop findings were triangulated with insights from ethnographic field notes, key informant interviews with stakeholders, focus group discussions with community members and document reviews. Data were coded manually and thematic content analysis was used to identify main issues. Results have shown that democratic selection of CAG members presented challenges in both urban and rural settings. We also noted that CAG members perceived their role as a form of employment which potentially led to ineffective representation of community interests. We conclude that democratic voting is not enough to ensure effective representation of community's interests of ethical relevance. CAG members’ abilities to understand research ethics, identify potential harms to community and communicate feedback to researchers is critical to optimise engagement of lay community and avoid tokenistic engagement.

## 1 BACKGROUND

Community engagement or public/patient involvement (PPI) is increasingly promoted in international research guidelines to protect communities from exploitation and harm. Studies in low income countries have shown that research presents a high risk of exploitation because some people may participate without full understanding of risks and benefits, since they are attracted to monetary incentives or health care.^1^ Engaging communities to advise on conduct of health research is therefore seen as a means of improving ethical research practice.^2^ Community engagement is also seen as helping to design research that responds to concerns in a community, improve trust, relevance, success and sustainability of interventions.^3^ There is however no widely agreed definition of community engagement and we employed the Council for International Organisations of Medical Sciences definition of community engagement because it offers ethical guidance on how to strengthen representation of communities in study design. We therefore define community engagement as:

*a process of engaging potential participants and communities in a meaningful participatory process that involves them in an early and sustained manner in the design, development, implementation, design of the informed consent process, monitoring of research and in the dissemination of its results*.^4^

While we concur that community members have capabilities to identify their needs and they should be actively involved in finding solutions to these needs,^5^ the degree of participation deserves critical attention. According to Sherry Arnstein, there are degrees of participation ranging from nonparticipation to citizen control.^6^ Nonparticipation occurs when communities are involved to be educated; tokenism occurs when they are informed or consulted but they lack power to influence decisions; while citizen control is attained when they are actively involved in planning, designing and have power to influence decisions.^7^ This implies that meaningful engagement occurs when all parties effectively participate in discussions to identify solutions. The feasibility of integrating both lay and scientific perspectives in research design however remains a challenge particularly in low literacy settings.

One of the approaches used to involve communities in health research is the use of a Community Advisory Board (CAB) or Community Advisory Group (CAG). Community Advisory Groups were initially introduced in HIV/AIDS research to strengthen the representation of people affected by or living with HIV/AIDS in research planning and implementation.^8^ Some donors now require establishment of a CAG, particularly in low resource settings, to provide community oversight on ethical conduct of health research.^9^ Roles of CAG include reviewing study protocols and informed consent forms, representing community concerns, advocating for the rights of research participants, consulting with potential research participants to provide advice, identifying research priorities, assisting in development of study materials, study design and implementation.^10^

While engaging a CAG is designed to strengthen community engagement, existing literature demonstrates challenges. Some of the challenges include limited understanding of health research, monetary expectations, dependence on researchers for finances, and lack of authority to influence decisions concerning research.^11^ These challenges have led to scepticism about the advisory roles of CAG members and concerns that their involvement is sometimes tokenistic or ‘window dressing’ to fulfil donor requirements.^12^

In this paper, we report findings from an ethnographic study in Malawi that seeks to understand the purpose, relevance and benefits of community engagement as seen by different stakeholders in research. We start by discussing approaches used to select the CAG members and how these affected their roles. We also discuss contextual factors in urban and rural settings that affected selection and functions of CAG members and community perceptions of the CAG.

Models of CAG vary in terms of both composition and selection processes. In relation to composition, CAG vary in terms of whether they represent the broad community or specific populations.^13^ Similarly to the term community engagement, there is no widely agreed definition of a community.^14^ As such, the term community can be externally defined to refer to: a group of people residing within a particular geographical location, a group of people with a common characteristic, identity or illness, or simply, a group of people residing within the immediate surroundings of a health facility. The ambiguity of the term ‘community’ therefore presents challenges on who should legitimately represent community's interests in health research.

Recommendations on composition of CAGs include having a group with equal numbers of representatives of the traditional authority, democratically elected residents and participant representatives;^15^ a group of community leaders or a group with equal numbers of representative residents and participant representatives.^16^ Selection approaches for CAG members also vary between contexts. A mix of purposive selection, elections and mixed methods approaches have been reported in the literature.^17^ One of the recommended approaches to choosing representatives is through democratic elections.^18^ Buchanan suggests that CAG members must be selected through democratic elections if they are to have authority to speak on behalf of the community.^19^

While recommendations on both CAG composition and selection relate to ideas of representation, the concept of representation is complex, particularly in governance of health research. The Oxford Dictionary defines representation as: *‘speaking or acting on behalf of someone or formal statements made to an authority’*.^20^ However, representation or representativeness may also mean possessing characteristics similar to a particular group.^21^ While professional certification may authorise an individual to represent scientific interests, it is debatable what should authorise CAG members to represent community interest. The diversity in communities and technical expertise required in health research make the question of whom and how should communities be represented difficult. Few publications have focused on the feasibility of different selection approaches and consequent effects on CAG members’ role of representing communities. The dual roles of CAG members in representing community interests to researchers and vice versa, as well as to balance their conflicting interests have also not been adequately covered in the literature. This paper therefore seeks to contribute to these knowledge gaps.

## 2 METHODOLOGY

### 2.1 Setting

This study was conducted in an urban and a rural district in southern Malawi, where the Malawi Liverpool Wellcome Trust Clinical Research Programme (MLW) is implementing medical research projects. Malawi has a population of 17,215,000, and a majority of people (84%) reside in rural areas.^22^ The literacy rate for adults above 15 years old is 75% and literacy rates are lower in rural areas.^23^ The country is faced with a huge disease burden and the leading causes of mortality are: HIV/AIDS, malaria, pneumonia, diarrhoea, Tuberculosis (TB) and non‐communicable diseases.^24^

MLW was established in 1995 and initially focused on conducting research on malaria. At the time the study was conducted in 2015, MLW had implemented over 50 research projects covering a broad range of research topics including: HIV/AIDS, TB, malaria, non‐communicable diseases and vaccines. A Science Communication department was established at MLW in 2008 to lead both programme wide and study specific engagement activities. Some of the public/community engagement activities run by this department include: managing CAGs, running science cafes, a science exhibition project, a weekly radio programme, and regular community sensitization meetings. Two CAGs were set up in 2009 in an urban and rural setting respectively where MLW was implementing research projects. Twenty six members were selected from six townships in the urban district in Blantyre and 48 members were selected from 39 villages in the rural district in Chikwawa. The roles of the CAG were to identify community concerns or potential harms and to feedback these to MLW researchers. A manual was developed by science communication staff which was used to guide selection, operation and training of CAGs. There were, however, no clear guidelines to determine the types of studies needing to engage a CAG. The decision to engage a CAG in a research project was therefore left optional to researchers.

CAG members were volunteers who resided in geographical locations where MLW was implementing research; CAG members from urban areas were selected from geographical locations surrounding health facilities which hosted various research projects. For rural areas, CAG members were selected from geographical locations where a community based intervention was being implemented. CAG members were trained by science communication staff on the following topics: MLW's vision, functions of CAG, clinical research, protection of research participants, leadership skills and report writing. MLW organized quarterly meetings for CAG members and the science communication team, where CAG members presented their reports and discussed new research projects as well as other ongoing research projects.

### 2.2 Data collection

The findings in this paper were part of doctoral research on community engagement in health research. Data collection took place between May 2015 and February 2016 after the CAGs had functioned for six years. Data collection included five different methods: 1) participatory workshop with CAG members, 2) document reviews, 3) participant observation among communities where medical research is conducted, 4) focus group discussions with community members who were not CAG members, and 5) interviews with research staff, CAG members, and community leaders. The study was approved by the University of Malawi, College of Medicine Research Ethics Committee in Malawi and Liverpool School of Tropical Medicine Research Ethics Committee in UK.

#### 2.2.1 Participatory workshops with CAG members and document reviews

We conducted two participatory workshops with CAG members from each district with an aim of understanding their roles in research. A total of 21 CAG members attended the workshop in the rural setting while a total of 16 CAG members attended the workshop in the urban setting (see Table 1). Workshop participants were purposively selected from a list of CAG members based on gender and geographical location. Both workshops were co‐facilitated by the first and third authors. Workshop participants were asked to fill a registration form and a summary of socio demographic details has been provided in Table 1. At the workshop, participants were asked to discuss how they were selected, their roles in research and more specifically who they represent and how they represent them. Responses were noted to understand how participants were selected as CAG members while group discussions were used to understand their roles in health research. Workshop participants also shared experiences of how they executed their responsibilities. Consent was sought from workshop participants to record workshop proceedings and each workshop lasted for about six hours. Following this, we carried out document reviews of past CAG meeting reports in order to understand concerns raised by the CAG members. Preliminary findings were presented to science communication staff for feedback.

**Table 1:** Socio demographic details of workshop participants

	Workshop‐rural	Workshop‐urban	Total
**Gender**			
Male	11	10	21
Female	10	6	16
**Age**			
20‐30	7	0	7
31‐40	5	6	11
41‐50	7	4	11
51‐60	2	3	5
61‐70	0	3	3
**Education**			
Primary education	15	4	19
Secondary education	6	5	11
Post secondary	0	7	7
**Profession**			
Business	0	3	3
Farmer	19	1	20
Community Health Worker	0	5	5

#### 2.2.2 Focus group discussions, interviews and participant observation

Main themes from the workshops, document reviews and discussions with science communication staff were further explored in subsequent data collection to broaden our understanding of the issues. We conducted eight focus group discussions with men and women from the two sites in order to explore diverse perspectives of how the CAG members functioned in the community. An additional 15 interviews were conducted with key informants to understand certain themes such as selection processes and roles of CAG members in more detail. These key informants were selected based on their involvement in selected research projects and included community leaders, CAG members, research participants and research staff. Topic guides developed from the workshop themes covering issues of selection processes, roles, communication, community concerns and community representation in research were used to facilitate interviews and focus group discussions.

Data from participatory workshops, interviews and FGDs were triangulated with field notes from observations. Participant observation was used to understand both explicit and unarticulated aspects of how the CAG members functioned in the communities. We participated in activities involving researchers, community engagement staff, field workers, CAG members and community members to observe their interactions and to become familiar with the context where they lived.

### 2.3 Data analysis

Workshop proceedings were transcribed by the lead author. The transcripts were read and coded manually based on the main issues arising from the discussions. Codes were later grouped into higher level themes of selection, motivation, roles, communication and feedback on research. Thematic analysis was used to compare and present discussions from the urban and rural CAG in relation to the main themes.

Interviews and focus group discussions were also recorded using a digital recorder and transcribed. A coding framework was developed by the lead author and transcripts were coded in QSR Nvivo 10. Main themes in relation to selection, roles of CAG and representation were used to support findings from the workshops. Findings were triangulated by using multiple data collection methods and crosschecking responses against various informants and the field notes. Results from this study were presented to CAG members for feedback in a separate workshop. We also sought their views on how to select CAG members in the future and empower them to effectively represent community's interests.

## 3 RESULTS

### 3.1 Challenges with selection of CAG members in urban and rural settings

MLW intended that CAG members would be selected democratically by community members residing in the geographical locations where research projects were being implemented. It was therefore expected that CAG members would reflect socioeconomic characteristics of the community. A democratic selection process required that community leaders would organize community meetings to elect CAG members and individuals would be nominated by fellow community members to participate in an electoral process. Every meeting attendee was asked to vote for their preferred candidate by a show of hands while facing down and the nominee who won the majority of votes would serve as a CAG member.

This election process proved more feasible in rural areas than urban areas. During the workshop, we asked workshop participants how they were selected as CAG members. The responses indicated differences between the urban and rural areas. Most of the workshop participants from rural areas stated that they were elected by fellow community members while a majority of workshop participants from urban areas indicated that they were selected by community leaders or health care workers as shown in the following quote: *‘I was chosen by the chief of the whole village to explain to people about research’* (Male, CAG member, urban setting). Workshop participants in both settings believed that they were selected because they were active in other community groups, knowledgeable about health issues or well known in their community.

Implementation of voting system for CAG members was easier in rural than in urban areas. This was because the villages or geographical locations in rural areas were small and communities were more homogeneous. Communities in rural areas were close‐knit, shared the same tribe and language, and demonstrated similar socio‐economic characteristics. In addition, people in rural areas were often long‐term residents in a particular village, familiar with one another and usually available during community meetings because they were mostly farmers. Since a majority of people from rural areas were available during community meetings and they were familiar with one another; this made it possible to nominate and vote for people they trusted to serve as CAG members. However, while community meetings were easy to organize in rural areas, the election process was not always free and fair. For instance, some community members reported that at times some community leaders influenced their followers to vote for people from their clans which clearly raise questions about democratic selection.

In contrast, the settings where research was being implemented in urban areas were larger and more densely populated. A majority of people in urban areas were originally from other parts of the country and had migrated to urban areas in search of employment. In addition, there was also high in and out migration. Communities in urban areas were therefore diverse and comprised of people with different professions and tribes. Community leaders in urban areas reported challenges in inviting people for meetings, and general unwillingness of community members to attend community meetings:

*Only few people come to attend community meetings; when they hear that researchers are coming for a meeting, most people do not show up, but when they hear that they will receive free stuff or food, they show up* (Male, village head, urban setting).

Participants in the FGD and interviews with community members (who were not part of CAGs but some were participants in research studies) reported challenges in attending community meetings due to other competing activities such as employment, businesses and other social activities:

*When it comes to issues of research, most people are so reluctant to attend meetings…people complain because they have numerous things to do…some go to work, others do business, they say time is money, for them to come and just listen [to researchers] they feel there is nothing to benefit* (Mother of a research participant, urban setting).

Lack of participation presented challenges in urban areas to select CAG members by democratic process because few people attended community meetings to vote. To fill the gaps in CAG membership, most participants from the urban district stated that they were selected by chiefs or health care workers.

### 3.2 CAG members’ roles in research

The intended role of CAG members to MLW Science Communication was to identify potential harms and represent community concerns to researchers. Some research staff, however, engaged CAG members to facilitate communication towards the communities and help in implementation of research.

When the workshop participants were asked to discuss their roles in health research, almost all workshop participants stated that they were the bridge between researchers and community members, as illustrated in the following quote: *‘I was chosen to be the eyes of health care workers, researchers and community members’* (Male workshop participant, urban setting). However, while some discussed this as a ‘two‐way bridge’, the focus was primarily on accountability to the researchers. CAG members defined their role as a form of employment or hierarchical duty where the orders came from above rather than below from the community as highlighted in the following quote: *‘The one who give us information to relay to others is the one that we listen to, they are like our bosses’* (Male, workshop participant, rural setting).

While the intended role of CAG members was to represent community concerns to MLW; we observed minor differences in the roles of CAG members from urban and rural locations suggesting that the roles of CAG members were shaped in response to the study design, practical demands from research staff and the social context they lived in. The CAG members from urban areas often mentioned communication roles whilst CAG members in rural areas mentioned that they assisted in the implementation of field work activities.

CAG members in the urban district explained that their primary role was in communication: they were informed about new research projects taking place at MLW in order to share this information with other community members and encourage their participation during community meetings. One member indicated that

*We were told that we are the bridge between researchers and community members to raise awareness of new research in the community and ensure that people are more receptive* (Female workshop participant, urban setting)

On the other hand, CAG members in the rural district indicated that their role was primarily to aide fieldwork: they were often requested by fieldworkers to accompany them to potential research participants’ homes and to ensure that research participants comply with research procedures. CAG members therefore perceived that their role was to facilitate implementation of research and ensure that research participants comply with research procedures as shown in the following quotes:

*…Staff [researchers] usually tell us in advance that they will visit our village and we have to look for people [potential research participants] to work with them and they do that for consecutive days* (Male workshop participant, rural setting).

This discrepancy between role intended by the science communication staff and role understood by CAG members was explained by the science communication staff to be a result of several factors. Science communication staff reported that some CAG members assumed communication and fieldwork roles without being instructed because they hoped to be considered for employment as field workers. This view also surfaced during interviews with CAG members. Some of the CAG members expressed disappointment with researchers for not considering them for fieldwork positions. In addition, science communication staff reported that one of the main concerns raised by CAG members pertained to increasing financial incentives for CAG members rather than reporting back issues from communities. This suggests that membership in CAGs may simply be seen as instruments to address issues of poverty and unemployment; or that CAGs may be set up for instrumental reasons, which in itself is not a problem, but compromises ideals around wider ethical concerns and democratic representation.

### 3.3 CAG members’ ability to perform their functions in urban and rural settings

We found two major constraints limiting CAG members’ roles in performing both the intended role of representing community concerns and the perceived role of communicating study information: community awareness of the CAG, and CAG members’ knowledge of the research that they were asked to report on.

We noted that community awareness of a CAG was essential to elicit concerns from community members. Most of the workshop participants in rural settings stated that community members were aware of them and approached them to report problems, seek advice and clarity on issues regarding research. This was seen to help demystify the research when information was obtained from fellow community members and enhanced trust and acceptability of research. CAG members believed that they were able to clear misconceptions which improved acceptability of research as illustrated in the following quote:

*We are able to clarify misconceptions in the villages because we have relationships with community members. For instance, there was a study in our community and people used to say that when they draw blood, they pay you back in exchange for the blood and we would say no, they are reimbursing you for transport to go to the clinic* (Workshop participant, rural setting)

In urban settings, however, we found that community members who participated in focus group discussions were not aware of the existence of a CAG. This obviously makes it difficult to relay community concerns to the researchers and could have been due to contextual factors discussed earlier, such as large geographical locations, dense population and lack of transparent selection approaches.

The second aspect that affected the CAG's ability to perform their role effectively had to do with communication of scientific procedures. While we noted that in many cases, most CAG members were not able to explain the concept of research accurately through the workshop and reports from previous meetings; we also noted that most CAG members had sometimes difficulties recalling detailed scientific information about the numerous studies that they were involved in. For instance, after presenting the aims of this research in the second workshop, workshop participants were asked to write down the purpose of this research project and what was going to happen. The majority did not give accurate information about the research project which again raises questions about how research would have been communicated to other community members. These issues show that CAG members’ perceived role in communicating research may have been compromised by inappropriate communication or overtly technical research procedures presented in non‐lay terminology.

These findings suggest that even though community members selected CAG members who were perceived as health literate, those selected as CAG members were sometimes unable to explain detailed research procedures to others. Overall, the discrepancies in expectations and CAG members’ difficulties to perform their role of informing communities about research lead us to question whether CAGs are an appropriate mechanism to represent community interests and ethical concerns.

## 4 DISCUSSION

This paper highlights practical experiences of selecting and engaging CAG in a low resource setting. Whilst democratic selection of community representatives is seen to strengthen the roles of CAG to represent community concerns in research,^25^ these results have shown that this is problematic across different settings. Despite using democratic selection, we noted that the CAGs did not reflect all relevant socio‐economic characteristics of the communities as intended. Since the CAG members were selected based on geographical location; the CAG did not include people who represented interests of other communities affected by the diseases being researched. In addition, rather than being a diverse group representing the demographics of the community, there were no members younger than 20 and a third of the members were community health workers in the urban setting. We also noted that most of the CAG members across both rural and urban areas (21 out of 37) had additional leadership roles in religious and other social groups; they were selected based on their perceived knowledge of health issues; for being known to others; or were preferentially put forward by powerful community members. Other studies have shown that selecting people from positions of authority may lead to choosing individuals whose outlook and interests are not in line with those from the most vulnerable groups.^26^ This leads us to question the idea that democratic selection would lead to socio‐demographic representation. Clearly, if socio‐demographic diversity is sought, extra measures need to be taken to recruit CAG members from these backgrounds.

These findings further suggest that despite selecting CAG members who were seen as ‘health literate and influential’ by some community members, CAGs were not effective in representing community interests of ethical relevance. Because of the limited evidence to demonstrate how CAG members contributed to reducing harm or exploitation of communities, we argue that use of CAG in facilitating communication between researchers and community can be categorised as tokenistic. The rationale for engaging communities is that the community stands to bear the risks or benefits of research and they have to be protected from harm and exploitation. CAGs provide a mechanism for community consultation on research design in order to minimise potential risks of research to participating communities.^27^ Our findings have, however, shown that the CAG members perceived that their main role was to facilitate communication and implementation of fieldwork activities which did not match participatory ideals in the literature. These findings are consistent with other research which showed that CAG or CAB members struggle to perform the expected roles of reducing exploitation during research but see their membership in CAGs as a form of (possible future) employment.^28^

Contrary to the challenges observed in our setting, other studies have reported successful experiences of CAGs providing a mechanism for community consultation.^29^ For instance, a study done by Morin in six study sites indicated that CAB members provided constructive feedback to improve the quality of research protocols.^30^ Similarly, a study from South Africa reported that CAB members contributed to minimizing exploitation to communities.^31^ Despite these few successful examples, challenges to engage communities appear to come down to power dynamics between researchers and CAG members, low science and ethics literacy, and limited access to resources independent from the research projects that they advise on.^32^

Our results suggest that neither democratic nor purposive selection approaches for CAG members led to effective representation of community concerns to reduce harm in research. Despite efforts to engage communities in research design, decision making was mostly done by researchers because of their expertise.^33^ We appreciate that researchers are trained and paid to conduct quality research and are accountable to funders but researchers’ obligation to conform to scientific procedures and international research ethics may render community representation ineffective if community feedback deviates from internationally acceptable research procedures. Given the findings from this study that CAG members did not provide a mechanism for collaborative partnerships between researchers and community, the question still remains if trying to establish genuine partnerships with communities using CAGs and share equal decision making power is desirable.

Since existing literature has shown that outcomes of similar models of CAG/CAB may vary across different contexts^34^; several questions remain unanswered on how to optimise the engagement of lay communities and avoid tokenistic engagement across different contexts. In our case, we presented these findings to CAG members and sought their feedback on how to address the challenges. In order to improve representation of community members, CAG members advised that researchers must map social groups in a given context and purposively identify individuals to serve as CAG members. Alternatively, the CAG members suggested using existing self‐organised community meetings in urban settings to elect CAG members. We however, acknowledge the limitations of these approaches in that they may not result in a representative group because youths, elderly people and other discriminated groups may be excluded.

Since we have shown that CAG members’ role in representing community concerns may be compromised due to their expectations of employment and financial incentives from researchers, we propose that CAGs must be independent. As such, funding for operations of CAG must be unrelated to the projects that they advise on, solicited from independent sources or channelled through independent community based organisations. In order to improve their effectiveness to represent community interests, terms of reference for CAGs should be jointly developed with elected CAG members specifying roles of CAGs and the basis for sharing equal decision‐making powers. Such terms of reference should also be made clear to field workers, research staff and community members to improve transparency and accountability. We also concur with other authors that capacity building for CAG members in research ethics, critical thinking and communication is essential for them to function effectively.^35^ Most importantly, CAG members should be engaged in determining potential harms to their community and their feedback should be incorporated in research design.

## 5 CONCLUSION

We used a qualitative study design to understand functions and effectiveness of CAGs as seen by research participants and community. Our findings indicate that democratic elections of CAG members were not entirely feasible and did not lead to selection of a CAG that reflected diverse community characteristics. Moreover, responses from CAG members failed to indicate how they addressed ethical concerns or discussed potential risks to study participants and communities. Primarily CAG members saw their roles as facilitating implementation of research which is contrary to the ethical mandate in the literature for CAGs.

Despite the challenges, we believe that having CAGs in place can uphold the requirements mandated by ethical guidelines and that communities should be engaged in a meaningful participatory process throughout study design. Further guidance and commitment is however required, on how to achieve this in order to ensure equal decision making power in collaborative partnerships and to avoid tokenistic engagement. We conclude that a non‐instrumental set‐up of a CAG, shared ideas about their roles in research, and CAG members’ understanding of scientific issues, research ethics and ability to communicate feedback aimed to reduce potential harm to communities are critical to meaningful participation.

## CONFLICT OF INTEREST

No conflicts declared.

## Notes

1 Emanuel EJ, Wendler D, Killen J, Grady C. What Makes Clinical Research in Developing Countries Ethical? The Benchmarks of Ethical Research. Journal of Infectious Diseases. 2004;189(5):930‐7; Geissler PW, Kelly A, Imoukhuede B, Pool R. He is now like a brother, I can even give him some blood’‐ Relational ethics and material exchanges in a malaria vaccine trial community in the Gambia. Social Science & Medicine. 2008;67:696–707; Massawe IS, Lusingu JP, Manongi RN. Community perception on biomedical research: A case study of malariometric survey in Korogwe District, Tanga Region, Tanzania. BMC Public Health. 2014;14(1):1–9; Mfutso‐Bengo J, Ndebele P, Jumbe V, Mkunthi M, Masiye F, Molyneux S, et al. Why do individuals agree to enrol in clinical trials? A qualitative study of health research participation in Blantyre, Malawi. Malawi Medical Journal. 2008;20(2):37.

2 Dickert N, Sugarman J. Ethical Goals of Community Consultation in Research. American Journal of Public Health. 2005;95(7):1123–7.

3 Mikesell L, Bromley E, Khodyakov D. Ethical Community‐Engaged Research: A Literature Review. American Journal of Public Health. 2013;103(12):7–14; Minkler M. Community‐based research partnerships: Challenges and opportunities. Journal of Urban Health : Bulletin of the New York Academy of Medicine. 2005;82(Suppl 2):3–12.

4 Council for International Organisations of Medical Sciences. International Ethical Guidelines for Health‐related Research involving Humans, Fourth Edition 2016 [cited 03 April, 2017]. Available from: http://cioms.ch/ethical-guidelines-2016/WEB-CIOMS-EthicalGuidelines.pdf.

5 Glassman M, Patton R. Capability Through Participatory Democracy: Sen, Freire, and Dewey. Educational Philosophy & Theory. 2014;46(12):1353–65.

6 Arnstein S. A Ladder of Citizen Participation. JAIP. 1969;35:216–24.

7 Ibid

8 Cox LE, Rouff JR, Svendsen KH, Markowitz M, Abrams DI. Community Advisory Boards: Their role in AIDS clinical trials. Health & Social Work. 1998;23(4):290–7.

9 Boulanger RF, Seidel S, Lessem E, et al. Engaging communities in Tuberculosis research. Lancet. 2013;13:540–5.

10 Morin SF, Maiorana A, Koester KA, Sheon NM, Richards TA. Community consultation in HIV prevention research: a study of community advisory boards at 6 research sites. Journal of Acquired Immune Deficiency Syndromes. 2003;33(4):513–20; Newman SD, Andrews JO, Magwood GS, Jenkins C, Cox MJ, Williamson DC. Community Advisory Boards in Community‐Based Participatory Research: A Synthesis of Best Processes. Preventing Chronic Disease. 2011;8(3):1; Quinn SC. Ethics in public health research: protecting human subjects: the role of community advisory boards. Health Policy and Ethics Forum. 2004;94(6); Strauss RP, Sengupta S, Quinn SC, et al. The Role of Community Advisory Boards: Involving Communities in the Informed Consent Process. American Journal of Public Health. 2001;91(12).

11 Cox LE et al. *op. cit*. note 8; Morin SF et al. *op. cit*. note 10; Manda‐Taylor L. Establishing community advisory boards for clinical trial research in Malawi: engendering ethical conduct in research. Malawi Medical Journal. 2013;25(4):96; Shubis K, Juma O, Sharifu R, Burgess B, Abdulla S. Challenges of establishing a Community Advisory Board (CAB) in a low‐income, low‐resource setting: Experiences from Bagamoyo, Tanzania. Health Research Policy and Systems. 2009:7; Pratt B, Lwin KM, Zion D, Nosten F, Loff B, Cheah PY. Exploitation and community engagement: Can community advisory boards successfully assume a role minimising exploitation in international research? Developing World Bioethics. 2015;15(1):18–26.

12 Quinn SC. *op. cit*. note 10.

13 Morin SF et al. *op. cit*. note 10; Shubis K et al. *op. cit*. note 11; Manda‐Taylor L *op. cit*. note 11; Pratt B et al. *op. cit*. note 11; Reddy P, Sifunda S, James S, Naidoo N, Buchanan D. The role of community advisory boards in health research: Divergent views in the South African experience. Sahara J. 2010;7(3):2–8.

14 Macqueen KM et al. What Is Community? An Evidence‐Based Definition for Participatory Public Health. 2001; Marsh VM, Kamuya DK, Parker MJ, Molyneux CS. Working with Concepts: The Role of Community in International Collaborative Biomedical Research. 2011; Tindana P, Singh J, Tracy S, et al. Grand Challenges in Global Health: Community Engagement in Research in Developing Countries PLoS Medicine. 2007;4(9).

15 Buchanan D, Sifunda S, Naidoo N. Assuring Adequate Protections in International Health Research: A Principled Justification and Practical Recommendations for the Role of Community Oversight. Public Health Ethics. 2008;1(3):246–57.

16 Ibid.

17 Ntshanga SP, Ngcobo PS, Mabaso MLH. Establishment of a Community Advisory Board (CAB) for tuberculosis control and research in the Inanda, Ntuzuma and KwaMashu (INK) area of KwaZulu‐Natal, South Africa. Health Policy. 2009;95(23):211–5; Pinto RM, Spector AY, Valera PA. Exploring group dynamics for integrating scientific and experiential knowledge in Community Advisory Boards for HIV research. AIDS Care. 2011;23(8):1006–13; Reddy P et al. *op. cit*. note 13; Shubis K et al. *op. cit*. note 11.

18 Brieland D. Community advisory boards and maximum feasible participation. American Journal of Public Health. 1971;61(2):292‐6; Buchanan D et al. op. cit. note 14.

19 Buchanan et al. *op. cit*. note 15, p. 7.

20 Oxford University Press. *Oxford Dictionaries Language Matters*. 2016 [Accessed 24 June 2016]; Available from: ttp://www.oxforddictionaries.com/definition/english/representation.

21 Brown MB. Science in democracy: expertise, institutions, and representation [electronic book]. Cambridge, MA : MIT Press, c2009.; 2009.

22 The World Bank. Malawi‐The Country at a Glance 2016 [updated 2016; cited 27 June, 2016]; Available from: http://www.worldbank.org/en/country/malawi

23 National Statistics Office & ICF Macro. Malawi Demographic and Health Survey 2010. Zomba, Malawi: NSO & ICF Macro; 2011.

24 SanJoaquin MA, Molyneux ME, Benjamin L, et al. Surveillance Programme of IN‐patients and Epidemiology (SPINE): Implementation of an Electronic Data Collection Tool within a Large Hospital in Malawi. PLoS Medicine. 2013;10(3); WHO. Malawi: WHO statistical profile. 2015 [updated 2015; cited 27 June, 2016]; Available from: http://www.who.int/gho/countries/mwi.pdf

25 Reddy P et al. *op. cit*. note 13.

26 Brieland D *op. cit* note 18; Kamuya DM, Marsh V, Kombe FK, Geissler PW, Molyneux SC. Engaging Communities to Strengthen Research Ethics in Low‐Income Settings: Selection and Perceptions of Members of a Network of Representatives in Coastal Kenya. Developing World Bioethics. 2013;13(1):10–20.

27 Quinn SC *op cit*. note 10; Strauss RP et al. *op cit*. note 10.

28 Lwin KM, Cheah PY, Cheah PK, et al. Motivations and perceptions of community advisory boards in the ethics of medical research: The case of the Thai‐Myanmar border. BMC Medical Ethics. 2014;15; Morin SF, Morfit S, Maiorana A, et al. Building community partnerships: case studies of Community Advisory Boards at research sites in Peru, Zimbabwe, and Thailand. Clinical Trials. 2008;5(2):147–56 10p; Pratt B et al. *op. cit*. note 11; Reddy P et al., *op. cit*. note 13.

29 Morin SF et al. *op. cit*. note 10; Pinto RM, Spector AY. *op. cit*. note 17; Reddy P et al. *op. cit*. note 13.

30 Morin SF, Maiorana A, Koester KA, Sheon NM, Richards TA. Community consultation in HIV prevention research: a study of community advisory boards at 6 research sites. Journal of Acquired Immune Deficiency Syndromes. 2003;33(4):513–20.

31 Reddy P, Sifunda S, James S, Naidoo N, Buchanan D. The role of community advisory boards in health research: Divergent views in the South African experience. Sahara J. 2010;7(3):2‐8.

32 Pratt B et al. *op. cit*. note 11; Brieland D *op. cit*. note 18.

33 Cargo M, Delormier T, Levesque L, Horn‐Miller K, McComber A, Macaulay AC. Can the democratic ideal of participatory research be achieved? An inside look at an academic indigenous community partnership. Health Education Research. 2008 October 1, 2008;23(5):904–14.

34 Shubis K et al. *op. cit*. note 11; Reddy P et al. *op. cit*. note 11; Ntshanga SP et al. *op. cit*. note 17; Lwin KM et al., *op. cit*. note 33.

35 Morin SF et al., *op. cit*. note 11; Pratt B et al., *op. cit*. note 11.

